# Coma Associated with Microscopy-Diagnosed *Plasmodium vivax*: A Prospective Study in Papua, Indonesia

**DOI:** 10.1371/journal.pntd.0001032

**Published:** 2011-06-07

**Authors:** Daniel A. Lampah, Tsin W. Yeo, Setiawan O. Hardianto, Emiliana Tjitra, Enny Kenangalem, Paulus Sugiarto, Ric N. Price, Nicholas M. Anstey

**Affiliations:** 1 National Institute of Health Research, Development-Menzies School of Health Research Research Program, District Ministry of Health, Timika, Papua, Indonesia; 2 Global Health Division, Menzies School of Health Research and Charles Darwin University, Darwin, Australia; 3 Division of Medicine, Royal Darwin Hospital, Darwin, Australia; 4 Mitra Masyrakat Hospital, Timika, Papua, Indonesia; 5 Department of Medicine, Sam Ratulangi University, Manado, Indonesia; 6 National Institute of Health Research and Development, Jakarta, Indonesia; 7 Nuffield Department of Clinical Medicine, Centre for Tropical Medicine, University of Oxford, Oxford, United Kingdom; University of California San Diego School of Medicine, United States of America

## Abstract

**Background:**

Coma complicates *Plasmodium falciparum* infection but is uncommonly associated with *P. vivax*. Most series of vivax coma have been retrospective and have not utilized molecular methods to exclude mixed infections with *P. falciparum.*

**Methods:**

We prospectively enrolled patients hospitalized in Timika, Indonesia, with a Glasgow Coma Score (GCS) ≤10 and *P. vivax* monoinfection on initial microscopy over a four year period. Hematological, biochemical, serological, radiological and cerebrospinal fluid (CSF) examinations were performed to identify other causes of coma. Repeat microscopy, antigen detection and polymerase chain reaction (PCR) were performed to exclude infections with other *Plasmodium* species.

**Results:**

Of 24 patients fulfilling enrolment criteria, 5 had clear evidence for other non-malarial etiologies. PCR demonstrated 10 mixed infections and 3 *P. falciparum* monoinfections. 6 (25%) patients had vivax monoinfection and no apparent alternative cause, with a median GCS of 9 (range 8–10) and a median coma duration of 42 (range 36–48) hours. CSF leukocyte counts were <10/ul (n = 3); 2 of the 3 patients without CSF examination recovered with antimalarial therapy alone. One patient had a tremor on discharge consistent with a post-malarial neurological syndrome. No patient had other organ dysfunction. The only death was associated with pure *P. falciparum* infection by PCR. Vivax monoinfection-associated risk of coma was estimated at 1 in 29,486 clinical vivax infections with no deaths. In comparison, the risk of falciparum-associated coma was estimated at 1 in 1,276 clinical infections with an 18.5% mortality rate.

**Conclusions:**

*P. vivax-*associated coma is rare, occurring 23 times less frequently than that seen with falciparum malaria, and is associated with a high proportion of non-malarial causes and mixed infections using PCR. The pathogenesis of coma associated with vivax malaria, particularly the role of comorbidities, is uncertain and requires further investigation.

## Author Summary

An estimated 132 to 391 million cases of *Plasmodium vivax* occur annually, accounting for up to 50% of malaria cases in South and East Asia. Vivax malaria is called “benign tertian malaria” and is not considered to be associated with life threatening or severe complications. Recently, observational studies and case series or reports have reported features of severe disease normally only seen in falciparum malaria, including coma. However, the majority of these reports did not exclude mixed infections using molecular methods or estimate the risk to the population in an endemic area. In Indonesia Papua, which has a high rate of vivax malaria and drug resistance, we prospectively enrolled all patients with coma and vivax monoinfection by microscopy over a 4 year period. We excluded falciparum malaria by molecular and antigenic methods, and sought evidence for other central nervous system (CNS) infections by cerebrospinal fluid microscopic examination. The majority of patients had evidence of mixed infections with *P. falciparum* or non-malarial causes of coma, however six patients had clinically significant coma, with no evidence of falciparum malaria or other CNS infections. The study was limited by the absence of bacterial cultures. Our study highlights that *P. vivax* can be associated with coma, but that this phenomenon is 23 times less frequent than that seen with falciparum malaria and was not associated with a fatal outcome.

## Introduction

Severe complications in malaria are normally associated with *Plasmodium falciparum* infections. Cerebral malaria, a serious manifestation of falciparum malaria is associated with a 10–40% mortality [1]. It is defined clinically as unarousable coma in the presence of any degree of *P. falciparum* parasitemia without evidence of other infectious or metabolic etiologies [2]. The central process in the pathogenesis of coma in falciparum malaria is thought to be microvascular obstruction resulting from cytoadherence of parasitized red cells to activated and dysfunctional endothelial cells [3], [4]. In contrast to *P. falciparum*, *P. vivax* has a low parasite biomass and until recently [5], was considered unable to cytoadhere or sequester [6]. While it has long been considered to be clinically uncomplicated, three recent large prospective epidemiological studies from Indonesia, Papua New Guinea and India have challenged this perception, associating *P. vivax* infections with severe manifestations of disease including anemia, respiratory distress, coma and death [7]–[9]. However, detailed clinical characterizations of specific complications, particularly coma, have not been described.

In addition to the epidemiological studies from Indonesia and PNG [7], [8], [10], 70 cases of *P. vivax* associated coma have been reported since 1921 [9], [11]–[28], the largest series being that of 12 patients from Russia published in 1943 [12]. In several reports, the clinical information provided was not detailed enough to exclude alternative diagnoses and the clinical presentations would not have been classified as cerebral malaria by WHO criteria [14], [19], [20]. Mixed infections with *P. falciparum* and *P. vivax* are not uncommon in severe malaria and can be notoriously difficult to diagnose [29]. Despite this the majority of the descriptions of vivax coma have relied solely on microscopy to establish the parasitological diagnosis, with modern methods to exclude mixed infections, eg polymerase chain reaction (PCR) or *P. falciparum* antigen detection, being applied to only 24 cases in 9 publications [9], [20], [21], [23]–[28].

To clinically characterize coma in *P. vivax* infections, we conducted a prospective observational study in Papua, Indonesia, of patients admitted with impaired consciousness and *P. vivax* monoinfection on the peripheral blood film, to confirm the diagnosis and investigate associated co-morbidities.

## Methods

### Study Site and Patient Population

The study was conducted at the Mitra Masyarakat Hospital, Timika, Papua, Indonesia, the only hospital in a lowland area with unstable transmission and a high prevalence of multi-drug resistant *P. falciparum* and *P. vivax* [30], [31]. The emergency department and wards were reviewed thrice daily. All patients with a Glasgow coma score of ≤10 at the time of examination by the research team, and a peripheral blood film reported as only *P. vivax* by the hospital laboratory were prospectively enrolled. On recruitment, history and physical examination findings were obtained using a standardized data form and patients followed daily until discharge. Oxygen saturation was measured with a pulse oximeter. All patients had venous blood samples collected for investigations including full blood counts (measured using a Coulter counter) and routine biochemistry. Acid-base parameters and lactate were analyzed using an iSTAT portable analyser (iSTAT Corp). Lumbar puncture was undertaken for clinical indications at the discretion of the treating physician, and if informed consent was obtained from patient's relatives. Plasma was also tested for antibodies to Murray Valley Encephalitis, Dengue, Kunjin and Japanese B Encephalitis viruses, using hemagglutination inhibition and indirect fluorescent antibody testing. All patients were treated with the recommended antimalarial treatment for severe malaria at RSMM Hospital, intravenous quinine until October 2005 and intravenous artesunate thereafter. Antibiotics were prescribed at the discretion of the treating physician who was independent of the study team.

### Microscopy, Polymerase Chain Reaction, Antigen Detection

All Giemsa-stained hospital slides at the time of enrolment were re-examined by a research microscopist with 15 years experience in malaria microscopy. These were considered negative for *P. falciparum* if none were seen on 100 high power fields. *P. falciparum* histidine rich protein 2 (HRP2) antigen was measured using the Paracheck (Orchid Biomedical Industries, Goa, India) rapid diagnostic test and *Plasmodium* species confirmed using a multiplex polymerase chain reaction [32].

### Cerebrospinal Fluid Examination

Where possible, cerebrospinal fluid (CSF) was collected, centrifuged and processed on the day of admission. CSF was Gram stained for bacteria and CSF white cells were quantitated manually using a Neubauer hemocytometer. Protein and glucose concentrations were measured by an autoanalyzer (Hitachi).

### Statistical Methods

Statistical analysis was performed with STATA software (version 9.2). Results are presented as either mean or median (range) for continuous variables, and proportions for variables with binary outcomes.

### Ethical Approval

Written informed consent was obtained from all patients' relatives, and ethical approval was obtained from the Health Research Ethics Committees of the National Institute of Health Research and Development, Indonesia and Menzies School of Health Research, Australia.

## Results

### Patients

**Figure 1:**
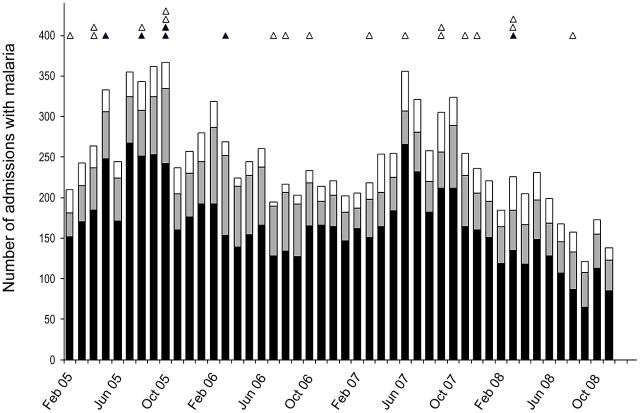
Number of cases of malaria admitted to RSMM each month from February 2005 to October 2008. Black areas on bars are *P. falciparum*, grey areas are *P. vivax* and white areas mixed infections. Black triangles are cases of vivax only on PCR. White triangles are cases of mixed or *P. falciparum* on repeat microscopy, PCR or antigen testing.

From February 2005 through November 2008, 40,160 patients were admitted to the hospital of which 29% (11,469) had a diagnosis of malaria (**Figure 1** **)**. *Plasmodium falciparum* infection was present in 7647 (67%) of these admissions, *P. vivax in* 2443 (21%), *P. malariae* 153 (2%), *P. ovale* 3, with 1223 (11%) mixed infections by routine hospital microscopy. Routine hospital surveillance identified a total of 246 patients admitted with coma and microscopy confirmed parasitemia; of these patients 174 (71%) had monoinfection with *P. falciparum,* 41 (17%) monoinfection with *P. vivax* and 31 (13%) with mixed infections. Of the 41 patients with *P. vivax* monoinfection by routine microscopy, 24 patients were admitted with coma (GCS ≤10) and were enrolled in the study.

Five patients were found to have alternative causes of coma based on clinical, radiological and cerebrospinal fluid findings: one with meningitis, two with head trauma and two children with severe pneumonia. On re-examination of the blood film and by antigen detection, five (20%) patients were found to be infected with both *P. falciparum* and *P. vivax*, with a median parasitaemia of 258/µL and 1273/µL respectively. These 5 mixed infections were confirmed by PCR analysis. PCR identified an additional 5 patients with mixed infections and 3 with *P. falciparum* monoinfection. In total, six (25%) patients had no evidence of mixed infections by microscopy, antigen and PCR testing and no clinical or laboratory evidence of an alternative diagnosis to account for the coma. The baseline characteristics, hematological and biochemical indices of all 24 patients are summarized in **Table 1** **.** Two of the six cases with PCR-confirmed *P. vivax* monoinfection were recruited during a two week period in October 2005 (**Figure 1** **)**.

**Table 1:** Baseline characteristics of patients.

	Coma with only *P. vivax* by PCR	Coma with only *P. vivax* on microscopy but PCR positive for mixed species or *P. falciparum*	Coma with *P. vivax* plus *P. falciparum* coinfection by microscopic cross- examination and/or HRP2 antigen positivity, or other diagnosis
Number	6	8	10
Age (yrs) Median and range	19 [10]-[25]	16 [6]–[30]	16 [1]–[30]
No of Males (% total)	4 (66%)	6 (75%)	8 (80%)
No of Indigenous Papuans (% total)	4 (83%)	6 (75%)	9 (90%)
Glasgow Coma Score Median [Range]	9 [8]–[10]	8 [5]–[11]	10 [4]–[11]
Time to recovery from coma (hrs) Median [Range]	42 [24–48]	48 [18–60]	84 [16–192]
Neurological sequelae	Yes	No	No
Fatal outcome (n)	0	1	0
Hemoglobin (g/dl) Median(Range)	11 (9.4–11.7)	10.1 (7.9–12.6)	10.8 (3.9–14.4)
Leukocytes (/µl) Median (Range)	9600 (8500–11400)	9250 (6800–13800)	10100 (4900–39600)
Platelets (x 10^3^/µl) Median (Range)	178 (133–221)	154 (74–377)	94 (32–328)
Glucose (mmol/L) Median (Range)	6.1 (4.8–7.8)	5.8 (4.675–8.3)	7.5 (4.8–217)
Lumbar Puncture Obtained	3/6 (50%)	6/8 (75%)	7/10 (70%)
CSF cells>50/µl	0/3 (0%)	0/6 (0%)	1/7 (14%)

Characteristics of the 6 individuals with confirmed pure *P. vivax* infection are detailed in **Table 2**. All were adolescents or young adults (aged 10–25) with a history of fever (median duration of fever prior to admission of 1 day (range: 1–7)), and a short history of decreased conscious level prior to presentation (1 hour [range: 0.5–2]). None of the patients had seizure activity reported prior to or during the hospital admission. None were reported to have had a previous history or a family history of neurological disease, or to have ingested medication (including antimalarials) or traditional medicines in the two months prior to presentation.

On examination all six patients were normotensive, with normal respiratory rate and oxygen saturation. The median Glasgow coma score (GCS) was 9 (range: 8–10). There were no focal or lateralizing neurological signs. Hepatosplenomegaly was present in two patients. There were no petechiae or purpuric lesions. Chest auscultation was normal. Chest x-rays were performed in 3 patients, none of which showed infiltrates.

**Table 2:** Clinical findings in the six patients with coma and PCR-confirmed monoinfection with *P. vivax*.

	Patient 1	Patient 2	Patient 3	Patient 4	Patient 5	Patient 6
Age (yrs)	20	18	15	25	10	19
Sex	Male	Male	Male	Male	Female	Female
Ethnicity	Non Papuan	Papuan	Papuan	Papuan	Papuan	Papuan
No of days with fever	3	1	1	7	1	14
Coma duration (prior to admission) hours	0.5	1	2.3	2	0.5	2
Convulsions (prior to admission)	None	None	None	None	None	None
Systolic Blood Pressure (mmHg)	110	111	110	120	116	110
Diastolic Blood Pressure (mmHg)	70	50	70	70	60	70
Respiratory Rate (/min)	24	25	24	24	26	28
Oxygen Saturation (%)	94	99	99	100	100	97
Temperature (^O^C)	37.0	39.1	36.8	36.0	38.7	36.7
Liver/Spleen palpable	No	Yes	No	No	No	Yes
Glasgow Coma Score (on enrolment)	8	8	10	9	10	8
Time to recover to GCS 15 (hrs)	48	36	36	48	48	48
Parasite Clearance (days)	2	2	1	1	1	1
Neurological Deficit	No	No	Yes	No	No	No
Anti-malarial Therapy	Artesunate	Artesunate	Artesunate	Artesunate	Artesunate	Artesunate
Antibiotics	No	No	Yes	Yes	Yes	Yes
Lumbar Puncture Obtained	No	No	Yes	Yes	Yes	No

### Biochemistry and Cerebrospinal Fluid

Peripheral blood parasite counts were low with a median of 212/µl (Range: 57 to 2131/µL) (**Table 3** **).** All patients were anemic, one had a slightly elevated leukocyte count (11,400/µl) but none had significant thrombocytopenia (**Table 3**)**.** Renal function was normal with no significant abnormalities in electrolytes or blood glucose concentration. No patient had significant acidosis (venous bicarbonate <15 meq/L), although two had elevated venous blood lactate levels (3.1 mmol/L and 5.1 mmol/L).

**Table 3:** Laboratory findings in the six patients with coma and PCR-confirmed monoinfection with *P. vivax*.

	Patient 1	Patient 2	Patient 3	Patient 4	Patient 5	Patient 6
Hemoglobin (g/L)	122	109	129	126	111	103
Leukocytes (/µl)	8500	9400	11400	10100	8700	9800
Platelets (x 10^3^/µl)	133	221	145	182	183	173
Sodium (mmol/L)	138	140	134	142	140	136
Potassium (mmol/L)	3.1	3.6	3.8	3.0	3.3	3.5
Chloride (mmol/L)	104	106	103	106	104	103
Glucose (mmol/L)	7.7	6.16	7.81	5.5	5.39	4.9
Creatinine	0.74	1.2	0.67	0.8	0.8	0.61
pH	7.43	7.37	7.44	7.45	7.40	ND
Bicarbonate (mmol/L)	27.5	17.6	23.8	20.6	23.5	ND
Base Excess	3	−8	0	3	−1	ND
Lactate (mmol/L)	1.28	5.13	1.9	3.1	ND	ND
CSF Cell Count (/µl)	ND	ND	7	3	7	ND
CSF Glucose (mg/dl)	ND	ND	64	90	53	ND
CSF Protein (mg/dl)	ND	ND	1	5	7	ND
Parasitemia (/µl)	213	94	57	656	2131	196
PCR	*P. vivax*	*P. vivax*	*P. vivax*	*P. vivax*	*P.vivax*	*P. vivax*

All values are for venous blood except where indicated.

Cerebrospinal fluid (CSF) was obtained from three patients; cell counts were all less than 10/µl, with a normal protein concentration and a CSF to blood glucose ratio >0.4 (**Table 2** **).** Acute plasma serology for flaviviruses did not show evidence of recent arbovirus infection in any patient.

### Treatment and Outcome

All six patients received intravenous artesunate, with four receiving additional antibiotics. The two patients without CSF examination recovered without antibiotic therapy. The median time for parasite clearance was 1.5 days (range: 1–2 days) with patients taking a median of 42 hrs (range: 36–48 hrs) to recover to a normal Glasgow coma score; **Table 2**. One patient had neurological sequelae after recovery from coma, consisting of an action and postural tremor with myoclonus (Video S1). Although improved, tremor/myoclonus was still present at the time of hospital discharge on day 5. The patient was subsequently lost to follow up. All patients with *P. vivax* monoinfection survived; the sole fatality was found in a patient with initial hospital microscopy showing *P. vivax* but a PCR result showing *P. falciparum* monoinfection.

### Population Risk

Over the 46 months of the study an estimated total of 176,915 clinical episodes of *P. vivax* were recorded in the community [31], and 2443 admitted to hospital. Assuming that 6 of the patients admitted with decreased conscious level were actually attributable to *P. vivax,* then the overall risk of vivax-attributable coma is estimated to be 1 in 29,486 clinical vivax infections in the community (incidence 3.4 per 100,000 [95%CI: 1.24–7.38]) and 1 in 407 vivax admissions to hospital (incidence 246 per 100,000 [95%CI: 90.2–533.8]). Comparably active surveillance of coma (GCS ≤10) associated with *Plasmodium falciparum* recorded 70 cases for the 16 month period from February 2005 to May 2006, with an estimated 89,375 cases of falciparum malaria in the community and 3,105 who were inpatients during the same time period. In comparison to vivax malaria, the risk of falciparum-associated coma was 1 in 1,276 cases in the community (incidence 78.3 per 100,000 [95%CI: 61.1–98.9]) and 1 in 44 hospital admissions (incidence 2,254 per 100,000 [95%CI: 1762–2840]). Estimates for both species are conservative, assuming that all patients with coma will have presented to the hospital. Mortality in falciparum-associated coma was 18.5% (13/70) compared to 0% (0/6) in vivax-associated coma (p = 0.2).

## Discussion

We report a prospective case series of 24 patients with cerebral malaria defined by World Health Organization criteria associated with *P. vivax* infection on admission microscopy. A high proportion of these had non-malarial causes of coma (21%), mixed-species infections (42%) or *P. falciparum* infection (12%). *P. vivax* was the only identified potential cause in 25% of these patients. Cerebrospinal fluid (CSF) findings in these patients were not consistent with other central nervous system infections, such as encephalitis. While microbiological laboratory resources were limited and culture facilities absent, the clinical, hematological and clinical biochemistry results did not suggest bacterial sepsis, or metabolic or toxic etiologies for the unarousable reversible coma. The absence of seizures prior to or during admission makes prolonged post-ictal coma unlikely.

Early reports of vivax associated coma included temperate countries such as Russia, Italy and the USA [12]–[14]; however since 1970, all other case reports and clinical series have come from India and Pakistan [9], [16]–[21], [23]–[28] with a single case report from Turkey [22]. With the exception of a recent prospective study from India [9], these have been retrospective case reports or series. Of the 70 cases reported, only 9 studies comprising 24 patients tested for mixed infections using either antigen detection or molecular methods. It is significant that one third of the patients in our study were diagnosed as having mixed or *P. falciparum* infections after PCR testing which had not been previously identified by microscopy cross-checking and antigen testing.

One patient developed a new postural and intention tremor with myoclonic jerks after recovery from coma, a presentation similar to post-malarial neurological complications reported after recovery from cerebral malaria due to *P. falciparum* [33]. Other neurological complications previously associated with *P. vivax* such as inflammatory demyelinating polyneuropathy and facial paralysis were not seen in our study [9]. The history of fever, lack of focal neurological signs, non-inflammatory cerebrospinal fluid and the time to recovery from coma are similar to the features of the coma seen in falciparum malaria [1], [2]. Unlike falciparum malaria [1], [2] and reports of vivax coma from India [9] we did not find other organ dysfunction such as acute renal failure, acute lung injury or hyperbilirubinemia. Acidosis and increased lactic acid concentrations related to the impaired microcirculatory flow are markers of severity in falciparum malaria [1]. In this study, no patient had significant acidosis as defined by WHO criteria for severe malaria, but two (33%) had elevated lactic acid levels, one markedly so. There were also no fatalities, in contrast to the mortality rates of 18.5% seen in falciparum-associated coma in Timika and the 15%-40% in cerebral malaria from *P. falciparum* reported elsewhere [1], although the small numbers do not allow a confident estimate of case-fatality rates in vivax-associated coma.

The results suggest that *P. vivax* infection may be a plausible, albeit rare cause of coma as well as the post-treatment neurological syndrome, although lack of microbiological facilities did not allow us to comprehensively exclude other infectious causes. In Africa, autopsy studies show that up to 23% of cases of cerebral malaria diagnosed by careful clinical definitions and *P. falciparum* peripheral parasitemia may be due to other etiologies [34]. The temporal clustering of several cases in October 2005 could be due to unidentified infectious etiologies being responsible for the fever and coma, with incidental *P. vivax* parasitemia. Limitations of our study include our inability to exclude viral encephalitis, although conclusive demonstration of a causative pathogen is also difficult in better resourced settings due to the limitations of current diagnostic technologies. However in those undergoing lumbar puncture, CSF findings did not suggest viral encephalitis. Furthermore, death or profound neurological sequelae, characteristic of endemic arboviral encephalitides in this region (eg Japanese B and Murray Valley Encephalitis viruses), were not seen. Other viral comas such as herpes simplex encephalitis are unlikely given the relatively short time to full recovery without specific anti-viral therapy. Lack of bacterial culture facilities did not allow us to exclude concurrent bacterial infections. Nevertheless, two of the five vivax coma patients recovered without antibiotic therapy and CSF findings did not suggest meningitis. We did not test for toxins which could cause coma, but all patients denied use of traditional or other medications prior to admission.

The pathogenesis of coma in falciparum malaria is not fully understood, and comorbidities are increasingly recognized as contributing factors even where coma is clearly attributable to *P. falciparum* infection [35]. Much less is known about potential mechanisms underlying coma in *P. vivax* [6]. In *P. falciparum* infection, cytoadherence of parasitized erythrocytes to endothelial adhesion receptors [3], and endothelial activation and dysfunction resulting from decreased nitric oxide bioavailability [4] contribute to increased parasite sequestration and impaired microcirculatory flow. *P. vivax* infected erythrocytes have been recently shown to cytoadhere to endothelial cells via chondroitin sulfate A and intercellular adhesion molecule 1 (ICAM-1), both expressed on brain endothelial cells, although at a frequency 10-fold less than *P. falciparum* [5]. This could account for the lower incidence of coma in vivax malaria although the degree of endothelial activation is greater in vivax than falciparum malaria [36]. Increased von Willebrand Factor (vWF) and decreased levels of the vWF cleaving protease, ADAMTS 13, have recently been described in uncomplicated vivax malaria and severe falciparum malaria [37], [38], and could contribute to microvascular obstruction. However, their role in the pathogenesis of severe vivax remains undefined. The lack of renal failure and significant thrombocytopenia or hemolytic anemia in vivax-associated coma does not suggest a thrombotic thrombocytopenic purpura-like syndrome due to altered vWF/ADAMTS13. Peripheral parasitemias in our series were all low, but whether this reflects a low biomass or is due to parasite sequestration or pooling elsewhere could not be determined.

A significant proportion of patients in our study were only diagnosed as having mixed infections by PCR, with negative results for *P. falciparum* on repeated microscopy and antigen detection testing. In these patients, the small *P. falciparum* parasite biomass should not result in clinical coma based on prevailing sequestration-based concepts of coma, and therefore one cannot exclude a contribution from *P. vivax* to coma in these patients.

Lung function studies suggest that pulmonary complications in vivax malaria may be related to pulmonary vascular accumulation of inflammatory cells with or without sequestration of *P. vivax-*parasitized erythrocytes [39]. A similar process might occur in cerebral malaria associated with *P. vivax* but has not been described. Sepsis-associated encephalopathy, observed in critically ill septic patients, has been associated with increased tumor necrosis factor (TNF-alpha) (32). Concentrations of TNF-α [40] and other pro-inflammatory cytokines [36] can be higher in vivax compared to falciparum malaria, and have been linked to vivax-associated disease severity in a series without central nervous system manifestations [41], however the role of inflammatory responses in vivax-associated coma are not known [6].

In summary, we describe a prospective case series of reversible coma associated with pure *P. vivax* infection with no overt clinical evidence for other central nervous system infections. All patients survived following treatment with intravenous artesunate; however one patient developed a tremor and myoclonus after recovery from coma, consistent with a post-malarial neurological syndrome previously described with falciparum cerebral malaria. Our study shows that compared to falciparum malaria, coma is a rare syndrome associated with pure vivax malaria, occurring in approximately 3.4 per 100,000 clinical episodes of infection. The role(s) of comorbidities and the pathogenic mechanisms underlying coma associated with *P. vivax* are not clear and warrant further investigation.

## Acknowledgments

We thank Govert Waramori, Ferryanto Chalfein, Prayoga, Tonia Woodberry for technical and logistical assistance; Marlini Malisan and Margaretha Ferre for nursing assistance, Mitra Masyarakat Hospital staff for clinical support; Mauritz Okeseray, Jeanne Rini Poespoprodjo, Lembaga Pengembangan Masyarakat Amungme Kamoro for support and assistance, and Nick Douglas for critical review of the manuscript.

## Supporting Information

Video S1 A patient with bilateral resting tremor which increased with movement after recovery from a coma associated with vivax malaria. The patient did not have similar symptoms prior to illness which is consistent with a post-malarial neurological syndrome.
(WMV)
